# Evaporation induced acoustic emissions in microfluidic vessels

**DOI:** 10.1098/rsos.231029

**Published:** 2023-12-13

**Authors:** S. Dutta, T. J. Bieling, G. J. Verbiest

**Affiliations:** Department of Precision and Microsystems Engineering, Faculty of 3ME, TU Delft, Mekelweg 2, Delft 2628CD, The Netherlands

**Keywords:** xylem, microfluidics, plant-acoustics, three-dimensional printing, evaporation, ultrasound

## Abstract

Fluid flow processes such as drainage and evaporation in porous media are crucial in geological and biological systems. The motion of the displacement front of a moving fluid through multi-phase interfaces is often associated with abrupt mechanical energy release, detectable as acoustic emissions (AEs). The exact origin of these pulses and their damping mechanisms are still subjects of debate. Here, we study the characteristics of such AEs during evaporation of water from artificial microfluidic vessels, inspired by the physiology of vascular water-transport in plants. From the extracted settling times of the recorded AEs, we identify three pulse types and attribute their origins to bubble formation, snap-off events and rapid pore invasion. We also show that the resonance frequencies between 10 and 70 kHz present in specific pulse types decrease with increasing vessel radius (ranging from 0.25 to 1.0 mm) and length (ranging from 2.5 to 10.0 mm). Our findings provide insight into evaporation-induced AEs from microfluidic systems, and their potential use in non-invasive inspection or vascular health monitoring.

## 1. Introduction

**Figure 1.**
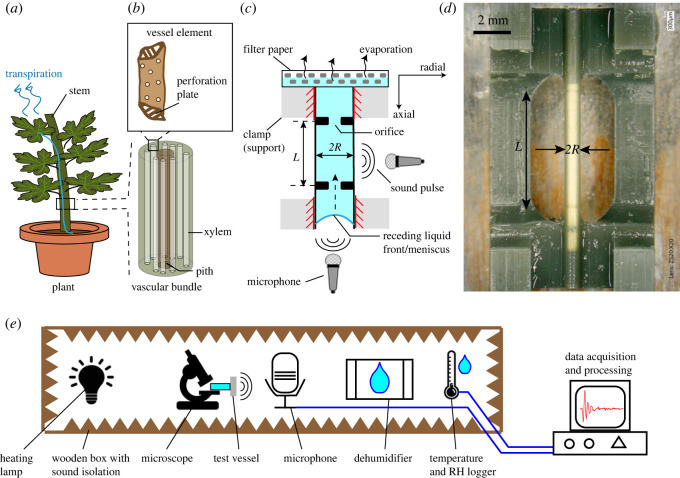
Motivation and method. (*a*) Schematic of a plant illustrating water flow via its tubular bundle of vessels terminating into the leaves. The diameter of these vessels in nature vary across orders of magnitude, from ∼1 µm until ∼100 µm, and can reach up to 700 µm [2,3]. Water evaporates through the stomatal pores of the leaves, creating a tension in the xylem vessels, which in turn leads to acoustic emissions (AEs) during release of stored elastic energy. (*b*) Schematic of a microscopic xylem vessel element. (*c*) Schematic of a water-filled three-dimensional printed vessel consisting of internal orifices (of thickness 0.3 mm) and clamped ends. Sound pulses emitted during drainage of water are recorded via the microphones. (*d*) Photograph of a fabricated vessel (*R* = 0.25 mm, *L* = 5 mm) under an optical microscope. (*e*) Schematic of the acoustic measurement chamber showing the heating lamp, microscope, test vessel, microphone, dehumidifier, temperature logger (RH, relative humidity), and the measurement computer.

Fluid flow in porous media and micro-conduits involve fluid–fluid interface displacement and complex interactions with the solid walls. Such constrained liquid movements are often associated with spontaneous acoustic emissions (AEs) [1], and even manifest in biological systems, e.g. AE during ascent of water via tubular xylem vessels in plants (figure 1*a,b*). Heavy evaporation from porous leaves, generates tension in the viscoleastic vessels that drives water-flow [4,5]. AE signals, produced in this process, have been linked to various possible origins [6–8], e.g. bubble nucleation, Haines jumps, snap-off events and elastic micro-movements in the grainy vessel walls, although a clear experimental evidence is lacking. Furthermore, AE under small values of tension (less than 100 kPa) have been also observed [1], but their features not well understood. AE monitoring holds a promise for non-invasive and data-rich exploration of pore-scale displacement processes of water. Recent works [9–13] studied the characteristics of ultrasound waveforms from drying plant shoots, and suggested a link between specific AE frequency content to an AE mechanism. The effect of mechanical resonances within xylem vessels on the AE characteristics was hypothesized in [13] and tested across plant species. However, a systematic study to these relationships in biological systems, such as plants, is challenging owing to their inherent concealed environment and lack of precise parameter control. Therefore, artificial structures that mimic the morphology and mechanics of natural vessels, could test hypotheses of the AE characteristics. Prior efforts in this direction include microchannels in poly(2-hydroxyethyl methacrylate) sheets [14], microchannel with porous evaporative sheets [15,16] and tubes connected to nanoporous ceramic discs [17].

These works emulated water-ascent in plants and studied the tension generated in the water column, however, it is not understood how these artificial structures impact the waveform and characteristics of the AE signals.

In this paper, we experimentally study the relationship between ultrasound pulse characteristics and geometry in three-dimensional-printed microfluidic vessels. These vessels mimic the dimensions and elasticity of water-carrying vessels in plants. We hypothesize that different mechanisms of AE generation have varying likelihood and that the resulting sound pulses may have distinguishable waveform characteristics. Inspired by prior works [10,13], we also hypothesize that the geometry of the vessels influence the resonance frequencies present in the externally recorded AE. To test these hypotheses, we induce evaporation-induced drainage of water in these artificial vessels, and show the presence of three types of sound pulses, characterized by their different ranges of pulse settling times (*τ*_s_). For each type, we determine their most likely physical origin. For the pulse type that we relate to the vessel geometry, we additionally study its frequency-domain characteristics and its dependency on vessel radius *R* and length *L*. Our study provides insight into non-invasive probing and diagnoses of tubular systems.

## 2. Methods

### 2.1. Fabrication

We fabricated the vessels using stereolithography based three-dimensional printing [18] with an EnvisionTEC Micro Plus Hi-Res 3D printer. Using digital light processing (DLP), a projector with pixel-size 30 µm was used to illuminate and harden specified locations on 50 µm thick layers of HTM140 V2 resin. The resin (hexane-1,6-diol diacrylate) has a Young's modulus *E* ≈ 1.56 GPa after hardening, as determined by uniaxial tensile loading (see the electronic supplementary material, and figure S3). In nature, the viscoelastic walls of xylem vessels in shrub-like species have similar moduli ranging from approximately 0.1 GPa to 10 GPa, depending on fibre density and the water content [19–21]. The printing resolution sets a constraint on the smallest dimensions of the printed vessels (radius *R* ∼ 150 µm). Too small vessel dimensions caused the resin to clog the channel [22]. The vessel structures were modelled in Fusion 360, sliced using the Perfactory RP software and printed. The final structure consists of a tube of which the outer two sides are clamped by a solid structure, as depicted in figure 1*c,d*. Orifices with a fixed thickness (≈ 0.3 mm) partition the vessel into vessel elements (analogous to perforation plates in xylem vessels). Vessels with varying *R* (0.25, 0.3, 0.5, 0.7, 0.9, 1 mm), for a fixed *L* = 5 mm, and with varying *L* (2.5, 3.0, 5.0, 7.0, 9.0, 10.0 mm) for a fixed *R* = 0.25 mm were fabricated. The chosen range of *R* is representative of that of xylem vessels in several shrub and tree species with wide vessels [2]. After printing, the vessels were rinsed in a Bandelin Sonorex RK31 ultrasonic bath containing 99% isopropyl alcohol as the solvent, and dried in air thereafter. Finally, the vessels were hardened by UV light (wavelength of 385 nm) curing (Dentalfarm Photopol Light Curing unit) for 3 min. In addition, a see-through vessel with identical design and a similar Young's modulus (approx. 1.84 GPa) was fabricated using a transparent 3DM Clear Tough resin in the same three-dimensional printer. This enabled us to observe the dynamics of the receding water-front (and associated weight loss) in a drying vessel under an optical microscope and identify acoustic events simultaneously.

### 2.2. Acoustic recording

We set up an acoustically isolated box to study the AEs from the drying vessels in a controlled and acoustic noise-free ambient (figure 1*e*). An incandescent 40 W light bulb was used to maintain a temperature between 30°C and 40°C in the box, and a Pingi dehumidifier was used to maintain the relative humidity to approximately 50%, thereby aiding evaporation from the vessels. A Lascar EL-USB-2 logged the temperature and relative humidity data. We observed the vessel under a portable USB powered optical microscope. We recorded AEs with two M500-USB microphones from Pettersson Elektronik AB (sampling rate of 500 kHz), placed along the axial and radial directions of the vessel. Figure 1*c*,*e* shows a schematic of the set-up including the measurement directions.

Prior to recording, the vessel is fully hydrated, and a wet filter paper (VWR grade 417) is placed at one open end of the vessel. The box is closed while we switch on the light bulb and start the acoustic recording with the microphone. Twenty-five minutes into the experiment, we switched off the light bulb as the temperature approached 40°C.

### 2.3. Data analyses

**Figure 2.**
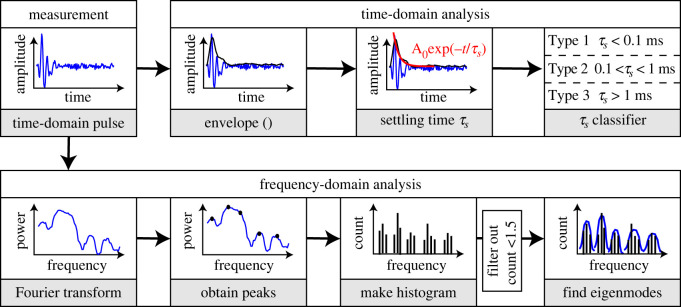
Flowchart of the waveform analysis used, starting with the time domain pulse, recorded by the microphones.

Figure 2 illustrates the data-analyses method. Axially and radially recorded pulses that occurred within 100 ms time offset from each other were selected. The choice for this time offset value was owing to the uncertainty in the synchronization of digital data acquisition between the two microphones. The maximum pulse amplitude and settling time were extracted in MATLAB (v. R2018b) from the time domain waveform using envelope detection followed by an exponential fit on the envelope. In frequency domain, the Welch power spectrum of the pulses was calculated using a Hanning window with a window size of 2500 samples. Subsequently, the power spectrum was smoothed using a moving average filter with a window of five samples. Peak frequencies were identified from individual spectra using findpeaks function with a minimum prominence of 5. Finally, the peaks of all the pulses from a given vessel were classified into a histogram with intervals of 3 kHz, where the mean frequency for each mode was assigned as the corresponding eigenfrequency of vibration.

### 2.4. Finite-element simulations

To gain insight into the origin of the mean peak frequencies, we used COMSOL Multiphysics to simulate the eigenmodes of the water-filled vessel structure with the appropriate geometry. We use a combination of the solid mechanics and pressure acoustics physics modules, which were coupled via the acoustic-structure boundary interaction. We used the eigenfrequency solver between 1 and 70 kHz to find the relevant modes of vibration. We modelled the vessel as an isotropic linear elastic material. A distributed sound-hard boundary was defined at the water–vessel interface, and a distribute sound-soft boundary was defined at the fluid–air interface (see the electronic supplementary material, figure S4A).

## 3. Results

### 3.1. Evaporation induced acoustic emission waveforms

**Figure 3.**
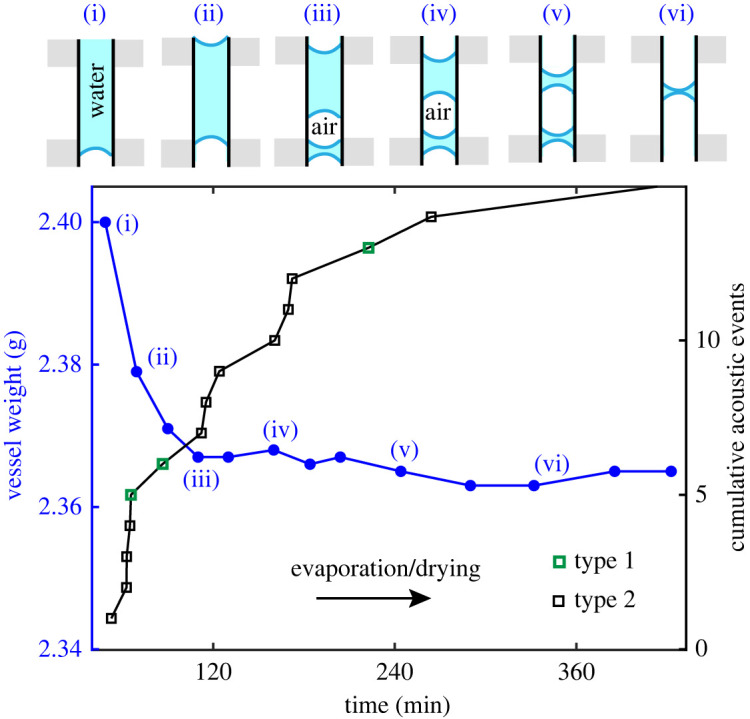
Measured weight (using a digital weighing scale of resolution 0.1 mg) of the water-filled vessel as a function of drainage/evaporation time and the cumulative recording (axial) of acoustic events. After 120 min, most of the water on the exterior surface of the vessel has evaporated, and subsequently the mass decreases slowly as the water meniscus recedes within the vessel, as schematically shown in the insets (i)–(vi). The AEs highlighted in green were observed to be of type 1, while the remaining AEs were of type 2. Based on the fluid front displacement visible on the camera during the evaporation experiment, the average speed of propagation was observed to be 1.36 mm h^−1^.

**Figure 4.**
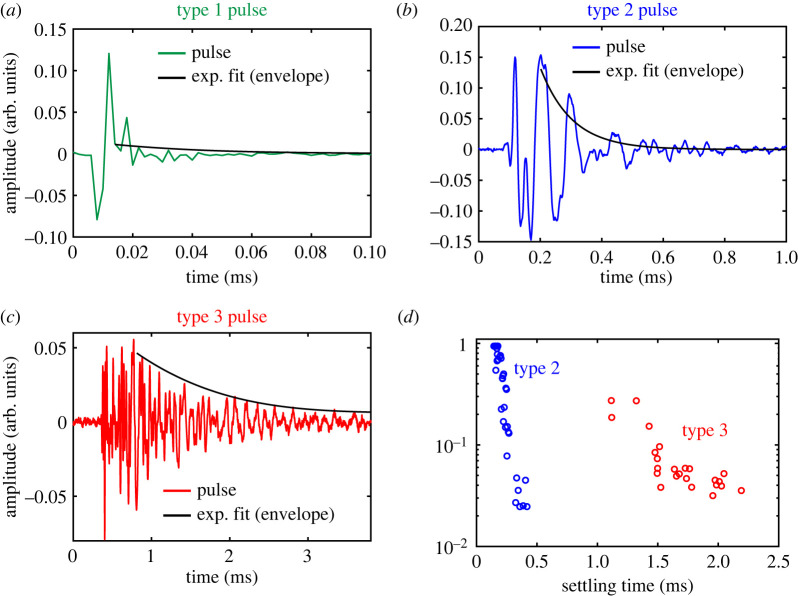
Figures (*a*) to (*c*) show an example of all three pulse types with their logarithmic fit, which we used to estimate the settling time. Figure (*d*) shows the settling time versus amplitude (logarithmic) for the type 2 and type 3 axial pulses. Type 1 pulses are not shown owing to their very sharp settling time. The pulses were recorded by the axial microphone for the vessel with *R* = 0.25 mm, and *L* = 5 mm.

Evaporation was induced on the test vessels. Figure 3 shows two regimes that were observed in the dynamic weight-loss curve for the vessel with length 5 mm and radius 0.25 mm. The first regime lasted for the initial 90 min and accounted for most of the recorded AEs. This indicates that evaporation from the surface and the filter paper were the most likely source of these AEs. The subsequent slower regime (receding water-column) was associated with the appearance of an air-front, that gradually displaces the water-front until the vessel has drained out (electronic supplementary material, figure S1A). One recorded AE event was synchronous with a fast snapping-off event of the water-front receding via the orifice inside the vessel (observed with optical microscopy), as shown in the electronic supplementary material, figure S1B. No significant differences in the waveform recorded axially were observed compared to that recorded radially (see the electronic supplementary material, figure S2A), indicating a common origin for both. Three types of pulses were identified based on significant differences in their *τ*_s_ as shown in figure 4*a–c*: type 1 pulses with *τ*_s_ < 50 µs, type 2 pulses with 0.1 ms < *τ*_s_ < 0.5 ms and type 3 pulses with *τ*_s_ > 1 ms. The envelope detection for the fast-decaying type 1 pulses was inaccurate owing to sampling rate limitation of the microphone. Clusters of amplitude versus τ_s_ of type 2 and type 3 pulses are shown in figure 4*d*.

### 3.2. Acoustic emission sources

**Figure 5.**
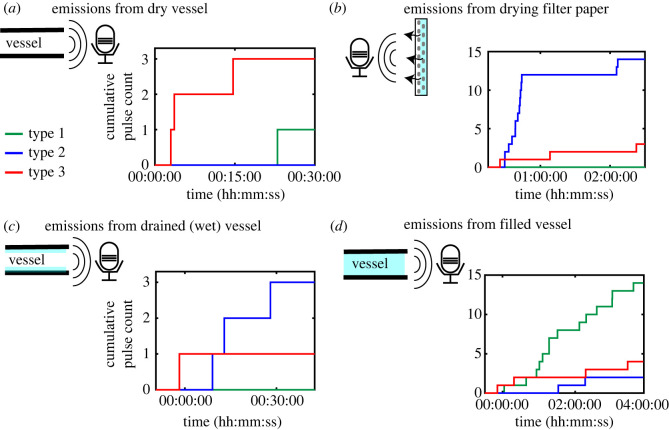
The four control experiments on sources of acoustic emissions: (*a*) heating a dry vessel, (*b*) the wet filter paper, (*c*) remnants of water in the vessel and (*d*) the vessel without heating or filter paper. For all experiments, we used a vessel with a length of 5 mm and a radius of 0.25 mm.

In order to identify the origin of different pulse types, we performed four control experiments. In experiment A (figure 5*a*), we studied a dry vessel without filter paper. The resulting pulses were mostly of type 3 with a long settling time. Their probable origin is the expansion of the three-dimensional printed material owing to the heating of the vessel. Emissions could be induced by micro-cracking or grain collision in the material, also observed in ceramics [23].

In experiment B, we recorded sound pulses from an evaporating filter paper, shown in figure 5*b*. Most of the acoustic pulses were emitted within 30 min, and most were of type 2. These acoustics are probably owing to Haines jumps, where the fluid inside the paper snaps back from the throat of one pore inside the filter paper to the next [24].

In experiment C, shown in figure 5*c*, we recorded from a drained vessel with remnant water adhering to the inner wall. A few pulses, mainly of type 2 were observed. Sources might include snapback of water and evaporation of retained water from tiny pores on the rough inner wall of the three-dimensional printed material.

In experiment D, we recorded pulses from a water-filled vessel without filter paper and without the light bulb heating the environment. This experiment, shown in figure 5*d*, shows that mostly type 1 pulses originate inside the vessel. Further, a few type 2 and type 3 events were also observed. Sources of the observed events could include snap-off processes, bubble-induced acoustics [1], Haines jumps [24] or snap-off processes in the drained part of the vessel, similar to that in experiments A, B and C.

### 3.3. Acoustic emission spectra and vessel parameter variation

**Figure 6.**
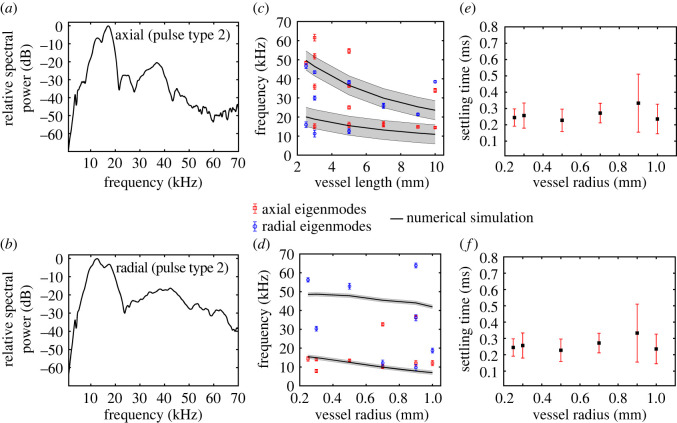
Mean and s.d. of extracted settling times of coupled type 2 pulses versus vessel radius, recorded in the (*a*) axial and (*b*) radial directions. Fourier spectra of example type 2 pulses recorded in the (*c*) axial and (*d*) radial directions. Extracted eigenmode frequencies using the peak detection method with varying (*e*) vessel length and (*f*) radius. Symbols represent the data from experiments and the solid curve represents the numerical simulation. For the numerical simulation, a Young's modulus of 0.78 GPa was used.

In order to investigate the frequency characteristics of the pulses, we computed their Fourier spectra (figure 2). Figure 6*a,b* shows, respectively, the fast Fourier transform (FFT) of representative pulses of type 2, recorded simultaneously along the axial and radial directions of the vessel with *L* = 5 mm and *R* = 0.25 mm. We observed characteristic peak frequencies of 17.2 kHz in the axial and 12.7 kHz in the radial direction, which we hypothesize to be governed by resonance modes of vibration. Type 2 pulses had the highest amplitude on average, and distinct peaks in the frequency domain between 5 and 70 kHz. Peaks with the highest amplitude lie between 10 and 20 kHz, while higher frequency peaks are also visible up to 60 kHz. The FFT of example type 1 and type 3 pulses are shown in the electronic supplementary material, figures 2C, and 2D. Since type 1 pulses occurred on a much faster time-scale, their spectra were very broad and featureless in the frequency range of interest. The frequency spectra of type 3 pulses were primarily of lower frequencies. Moreover, they featured several closely spaced peaks in the frequency range of interest. Therefore, we focus, in the next paragraph, on the peak frequencies of type 2 pulses, observe their dependencies on the vessel geometry and validate them with finite-element eigenfrequency simulations.

Figure 6*c* shows the extracted mean peak frequencies (with the highest significance) from the Fourier spectra against varying vessel length *L*. For each *L*, multiple peak frequencies are observed. We observe a decrease in frequency with increasing vessel length. Figure 6*d* shows the extracted mean peak frequencies against varying vessel radius *R*, where we observe a similar trend of decreasing peak-frequencies with increasing *R*. The error bars in the figures represent the standard deviation in each mean peak frequency, obtained from the histogram of several pulses as described in §2.3.

To gain insight into the origin of the mean peak frequencies, we used COMSOL Multiphysics to simulate the eigenmodes of the water-filled vessel structure with the appropriate geometry, as described in §2.4. Different vibration modes were observed namely, flexural, breathing, and modes owing to the orifices. Example mode-shapes are shown in the electronic supplementary material, figures 4B,C and 5E,F. Within the frequency range of interest, we observed that the orifice vibration modes (see black lines in figure 6*c,d*) show a dependence on *L* and *R*, that is in good agreement with those extracted from the measurements.

We further estimated the effect of fabrication tolerances on the simulated eigenfrequencies. In the fabrication, we observed a deviation of about 6% from the modelled geometry. As such, we assumed a conservative geometrical deviation of 10% in the simulations to obtain the sensitivity of the frequencies to a small variations in geometry. The highest sensitivity is on variations in *L*, where a deviation of up to 3 kHz in the obtained frequencies was observed. We used this value as the area within which we search for the calculation of the mean and standard deviation of the modes, as explained above. The sensitivity to *R*-variations was negligible.

In order to probe the probable damping mechanism in the acoustic events, we extracted the settling time (*τ*_s_) of type 2 pulses using an exponential fit on the pulse envelope. Figure 6*e,f* shows the mean and standard deviation in *τ*_s_ from coupled pulses recorded along the axial and radial directions, respectively. No significant dependence on the vessel radius was observed. The mean and standard deviation for type 2 pulses of the reference vessel were obtained to be 0.24 ± 0.08 ms.

## 4. Discussion

### 4.1. Waveforms and acoustic sources

We identified three types of waveform from the temporal signature of the acoustic pulses. The observation of distinct clusters in the amplitude-settling time plot, indicates the presence of pulses owing to multiple sources. Type 1 pulses were mainly observed in experiment D (figure 5*d*), which links their origin to the vessel interior via snap-off processes in the water column and the water–wall interface, or micro-bubble formations. These perturbations excite waves similar to the water-hammer phenomenon [25]. The majority of type 2 pulses occurred in experiments B and C (figure 5*b,c*), in the first 30 min of the filter paper drying. Some type 2 pulses occurred while water remnants evaporated from the vessel. Thus, these pulses originate from Haines jumps like events in the pores of the filter paper that further propagate along the vessel. The source of type 3 events is probably owing to microscopic grain collision induced thermally (experiment A) in the three dimensional printed material. Finally, we observed how a significant amount of pulses are correlated to the orifice modes seen in COMSOL. In these cases, the relaxation of surface tension in either the filter paper pores or imperfections in the three-dimensional printed material excites the orifices leading to the modes we observe.

The simultaneous optical and acoustic measurements showed drainage effects and accompanying acoustics. Drainage of the vessel occurred as follows: the fluid front recedes from the microphone side to the filter paper until the orifice traps it temporarily. Here it gathers enough tension to continue draining and then fully drains the vessel after passing the next orifice. We could not observe bubbles directly, either owing to their small size or their early merger with the receding fluid front. When they did appear, the fluid front subsequently entrained them. Similar observations were reported by Shi *et al*. [17] owing to improper charging of the vessels. We found an optical event synchronized with an AE of type 3 (see the electronic supplementary material, figure S1). Here the fluid front snaps off at the orifice, leading to the acoustic pulse.

Cavitation, as observed in plants, is unlikely to occur in the fabricated vessels, because the tension in the water column was not high enough to cause cavitation [17]. The Young–Laplace pressure (equation (1.1) in the electronic supplementary material) in the filter paper was estimated to be around 7.3 kPa, assuming a particle retention size of 40.00 µm, room temperature and pressure, and using an ideal contact angle of zero degrees. This upper bound estimate is significantly lower than the cavitation threshold of approximately 1 MPa [14]. Thus, smaller pore radii are needed to induce cavitation in further studies, e.g. nanoporous ceramic discs [17] or nanoporous anodic aluminium oxide membranes and hydrogel [26].

### 4.2. Acoustic emission spectra and vessel-geometry variation

Irrespective of the place of origin, the acoustic events propagate through the vessel and are, therefore, expected to be conditioned by the vascular geometry and mechanics. While acoustic damping can occur owing to viscoelasticity in the material or owing to multi-phase interfaces, the spectral content are likely to be influenced by the vessel dimensions. Numerical eigenfrequency analyses on the vessel structure have shown that the eigenmodes present in the most likely occurring pulse-type (type 2) can be attributed to vibrations of the orifice inside the vessel. In both simulations and experiments, we observed similar dependence of the eigenfrequencies on *L* and *R*. The discrepancies between simulation and experiment could be owing to multiple factors. Firstly, because of the statistical nature of the various acoustic events, we identified significant resonance modes based on the peak prominence (§2.3). As a result, the possibility of missing closely-spaced peak frequencies in datasets from different experiments on the same vessel cannot be entirely ruled out. Secondly, we modelled Young's modulus in simulation as that of an isotropic material while, in reality, DLP printed structures are highly anisotropic [27], thus we expect some margin of error.

In a prior work by Dutta *et al*. [13], an analytical relationship was derived between *R* and *τ_s_* for a fluid-filled acoustic resonator, given below:

4.1 $$R=\frac{4\eta_{l}\tau_{s}}{\rho_{l}},$$

where *η_l_* represents the fluid viscosity, and *ρ_l_* the density of the fluid. Applying equation (4.1) to the type 2 pulses in this work, the effective mean acoustic radius was obtained as 31.6 µm, which is close to the pore radius of the filter paper (approx. 40 µm). This suggests that the pores in the filter paper probably dominate the damping mechanism (acting as microscopic Helmholtz resonators), and therefore determining the observed *τ_s_*. Further, no significant variation in *τ_s_* with *R* was observed. This further supports the filter paper pores as the origin of the type 2 events in the experiments.

### 4.3. Impact and scope

This work sheds light on the characteristics and possible origins of spontaneous AEs during evaporation and drainage of microscopic vessels. The experiments show that such acoustic events can have multiple sources, even below cavitation threshold, and can be classified based on their time and frequency domain characteristics. The study opens a new route to systematically investigate AEs from analogous systems in biology and microfluidics. For example, a rapid ascent of water via xylem vessels driven by heavy transpiration in plants under drought-like conditions are known to cause bubble nucleation leading to AE. Detection and modelling of such phenomena can be useful to devise preventive measures against vessel blockage and plant mortality [7,8,11,28–30]. Our work employs three-dimensional printing to mimic the individual plant vessels. Although the printing material is a good representation of Young's modulus of a wide variety of plant vessels, the stereo-lithography technique has its limitations in covering the wide range of vessel/orifice radius, aspect (*L/R*) ratio, and viscoelasticity, of naturally occurring plant vessels. Further, the walls of plant xylem vessels differ in chemical composition (cellulose, pectin, lignin) from the resin used in three-dimensional printing, and contain bordering pit membranes. These features are hard to reproduce in artificial vessels and may affect natural AE characteristics. Advanced techniques like 2-photon polymerization [31], fibre spinning and extrusion [32] can be used to fabricate structures with smaller *R* and larger *L*/*R* ratio. Alternative materials like UV-cured hydrogels [33] can be used to extend the range of visco-elasticity and vessel mass density. Replacing evaporating medium with other liquids, e.g. saline or sucrose solutions, solutions with chemical composition similar to xylem sap, alcohols or volatile oils can be used to further investigate the impact of kinematic viscosity and surface tension on the AE signatures. Further, time-domain (transient) simulations could be performed to study the origin of other pulse types and their respective dependencies on the structural properties of the vessel.

The relationship between the acoustic pulses and mechanical resonances of the vessels opens the route of future studies on pulse transmission spectroscopy via external transducers. This provides a non-invasive way for inspection of viscoelastic properties of vascular networks. The same philosophy also holds for monitoring tubes in microfluidic systems and pipe flow engineering [34]. In microfluidics, acoustics could allow for the detection of fluid front displacement [24], and possibly even microdroplet size measurements similar to Lee *et al*. [35]. In pipe flow engineering, acoustics could allow for detecting fatigue cracks [36] and leaks [37] before they cause significant problems.

## 5. Conclusion

We analysed evaporation-induced AEs from plant-inspired artificial microfluidic vessels fabricated via three-dimensional printing. Using a porous filter paper as vessel termination, we induced evaporation on these vessels of varying dimensions and recorded acoustic pulses in a controlled environment. We categorized the pulses into three types based on their settling time and amplitude. By monitoring the occurence of each pulse type in control experiments, we identified their likely sources; these include fluid front displacement, material expansion and Haines jumps during evaporation of water from porous surfaces. Further, we extracted the characteristic peak frequencies in these pulses in frequency domain, which we attributed to eigenmodes of acoustic vibrations in the vessel and validated that by numerical simulations. The frequencies decreased with increasing vessel length and radius. The results provide a better understanding of evaporation induced acoustics in microfluidic vessels and their subsequent damping by the vessel. This could be beneficial in non-invasive probing of similar multi-scale tubular systems, both natural and artificial, e.g. plant physiology, microfluidics and geology.

## Data Availability

Data available from the Dryad Digital Repository: doi:10.5061/dryad.cvdncjt8n [38]. Supplementary material is available online [39].
